# Screening of protozoan and microsporidian parasites in feces of great cormorant (*Phalacrocorax carbo*)

**DOI:** 10.1007/s11356-017-8652-y

**Published:** 2017-03-02

**Authors:** Piotr Rzymski, Anna Słodkowicz-Kowalska, Piotr Klimaszyk, Piotr Solarczyk, Barbara Poniedziałek

**Affiliations:** 1 Department of Environmental Medicine, Faculty of Health Sciences, Poznan University of Medical Sciences, Poznań, Poland; 2 Department of Biology and Medical Parasitology, Faculty of Medicine I, Poznan University of Medical Sciences, Poznań, Poland; 3 Department of Water Protection, Faculty of Biology, Adam Mickiewicz University, Poznań, Poland

**Keywords:** Cormorants, Bird feces, *Cryptosporidium*, *Blastocystis*, *Microsporidia*, Microbial dispersion

## Abstract

The global population of great cormorants (*Phalacrocorax carbo* L.) is on the rise. These birds, characterized by rapid metabolism, can deposit large quantities of feces, and because they breed on the land but forage on water, both terrestrial and aquatic environments can be simultaneously affected by their activities. The contribution of great cormorants in the dispersal of bacterial and viral pathogens has been immensely studied; whereas, the occurrence of eukaryotic parasites such as protozoans and microsporidians in these birds is little known. The present study investigated the presence of dispersive stages of potentially zoonotic protozoans belonging to the genera *Blastocystis*, *Giardia* and *Cryptosporidium*, and *Microsporidia* spores in feces collected from birds inhabiting the breeding colony established at one lake island in Poland, Europe. The feces were examined by coprological techniques (staining with iron hematoxylin, Ziehl-Neelsen, and modified Weber’s chromotrope 2R–based trichrome), and with immunofluorescence antibody MERIFLUOR *Cryptosporidium/Giardia* assay. As found, the *Cryptosporidium* oocysts were identified rarely in 8% of samples (2/25; 3–5 × 10^3^/g) and no cysts of *Giardia* and *Blastocystis* were detected. Microsporidian spores were detected in 4% of samples (1/25) but at very high frequency (4.3 × 10^4^/g). No dispersive stages of parasites were identified in water samples collected from the littoral area near the colony. Despite the profuse defecation of cormorants, their role in the dispersion of the investigated parasites may not be as high as hypothesized.

## Introduction

The global population of great cormorant (*Phalacrocorax carbo* L.) is on the systematic rise, particularly in some parts of Europe where the number of breeding pairs is estimated to exceed 400,000 (Bregnballe et al. 2014; Klimaszyk and Rzymski 2016). This bird species, exterminated for decades by humans, have become numerous not only due to international and national law enforcements but also as a result of its high ecological adaptation, ability to forage on marine and freshwater environments, lack of regular predators, and increase in fish biomass due to the eutrophication and climate changes (Cramp and Simmons 1997; White et al. 2011; Skov 2011; Klimaszyk and Rzymski 2016). As these birds are generally gregarious, appear collectively, gather in flocks, nest in colonies on land, can feed on a relatively large area (up to 30 km from the colony), and simultaneously on various water systems, they may represent a significant biological factor that could trigger environmental modifications (Klimaszyk et al. 2015a; Klimaszyk and Rzymski 2016).

The effect of *P. carbo* on nutrient cycling, soil chemistry, terrestrial and aquatic vegetation, excessive algae growth (Ligęza and Smal 2003; Klimaszyk et al. 2015a, b), invertebrate communities (Kolb et al. 2010), and fish populations (Ostman et al. 2013) has been studied extensively (for review see Klimaszyk and Rzymski 2016). Some studies also addressed the role of these birds in dispersion of bacterial pathogens such as *Escherichia coli* (Klimaszyk 2012; Klimaszyk and Rzymski 2013a, 2016), avian influenza virus (Albini et al. 2014), avian paramyxovirus (Schelling et al. 1999), and West Nile virus (Iashkulov et al. 2008; Table 1). Recent studies also investigated the presence of gastric nematodes in these birds (Dziekońska-Rynko and Rokicki 2008; El-Dakhly et al. 2012).

**Table 1:** The current state of knowledge on the human pathogens dispersed by great cormorant (*Phalacrocorax carbo*)

Detected pathogen	Place of identification	Notes	Potential health threats	References
Bacteria				
*Escherichia coli*	Poland, Czech Republic (as a intestinal commensal it is spread anywhere the cormorant is present)	O25b-ST131 clone was isolated. The increased *E. coli* counts were observed in lake littoral and groundwater within the colony area	Predominantly serious urinary tract infections (O25b-ST131)	Tausova et al. 2012; Klimaszyk 2012; Klimaszyk and Rzymski 2013a
*Salmonella typhimurium*	Switzerland	Low prevalence	Serious gastroenteritis	Albini et al. 2014
Viruses				
Avian influenza virus H5	North-western area of the Caspian Sea	Very low prevalence	Highly pathogenic avian influenza transmitted between birds and to mammals resulting in death	Iashkulov et al. 2008
Avian paramyxovirus serotype-1	North-western area of the Caspian Sea, France (antibodies), Switzerland (antibodies)		Newcastle disease in poultry and wild birds. Clinical symptoms in human	Schelling et al. 1999; Artois et al. 2002; Iashkulov et al. 2008
West Nile virus	North-western area of the Caspian Sea	Mosquitoes are prime vectors, birds are main hosts	West Nile fever. Rarely neurological symptoms	Iashkulov et al. 2008
Fungi				
Microsporidia	Slovakia, Poland	*Encephalitozoon cuniculi* was identified (Slovakia).	Intestinal parasitosis, diarrhea	Malčeková et al. 2013; This study
Protozoan parasites				
*Cryptosporidium* sp.	Hungary, Netherlands, Poland	The exact genotype was not determined	Intestinal parasitosis, diarrhea	Medema 1999; Plutzer and Tomor 2009; This study
*Giardia* sp.	Hungary	The exact genotype was not determined	Intestinal parasitosis, diarrhea	Plutzer and Tomor 2009

The occurrence of dispersive stages of intestinal protozoan parasites such as *Giardia* cysts and *Cryptosporidium* oocysts in great cormorants is, however, largely unknown and, so far, reported only in two studies examining the bird feces (Medema 1999; Plutzer and Tomor 2009). The parasites were detected in cormorant droppings, but due to low number of samples in both studies, the definite conclusions on the role of cormorants in dispersion of these potential pathogens cannot be drawn. The presence of microsporidian spores in great cormorant, on the other hand, was so far a subject to only one study conducted recently in Slovakia. The spores, identified molecularly as *Encephalitozoon cuniculi* were detected using PCR in several fecal samples (Malčeková et al. 2013).

As some microsporidian and protozoan parasites are potentially infectious in mammals including human (Ehsan et al. 2015), it is of high priority to conduct further studies elucidating the role of great cormorants in their dissemination. As these birds represent a very important intermediate link in some food webs (Gwiazda et al. 2010, Skov et al. 2014) and a factor facilitating the dislocation of matter between terrestrial and aquatic ecosystems (Marion et al. 1994), it can be rather anticipated that they could also be responsible for high dispersion of parasites because they consume relatively large fish biomass, estimated at 350 g per day (Carss 1997). Various fish species were, in turn, identified as potential reservoirs of protozoan intestinal parasites such as *Giardia* sp. (Yang et al. 2010; Ghoneim et al. 2012) or *Cryptosporidium* sp. (Barugahare et al. 2011; Gabor et al. 2011) as well as microsporidian parasites (Lom and Nilsen 2003). Moreover, cormorants are characterized by rapid metabolism and the birds defecate on average 30 g dry weight of droppings per day (Marion et al. 1994). Deposited on relatively small area of colony, their chemical and microbial content can be subsequently transported with surface runoff and/or groundwater to the nearby lake (Klimaszyk and Rzymski 2011, 2013b; Klimaszyk et al. 2015a, b). Therefore, it is of great interest to evaluate the importance of great cormorants as vectors of dispersive stages of intestinal protozoan parasites in terrestrial and aquatic environments.

The present study aimed to investigate the presence of *Cryptosporidium* oocysts, cysts of *Giardia* and *Blastocystis*, and microsporidian spores in fecal samples collected from the colony of *P. carbo* during the breeding season. The colony, constituted of 170 breeding pairs, was located on the island of recreationally used, eutrophic Lake Chrzypsko (Northern Poland, Europe). To the best of our knowledge, this is the first study not only to survey such number of these birds in this regard but also to highlight that the role of great cormorants in dispersion of human intestinal protozoan and microsporidian parasites may not be as significant as expected.

## Material and methods

### The cormorant colony

**Fig. 1:**
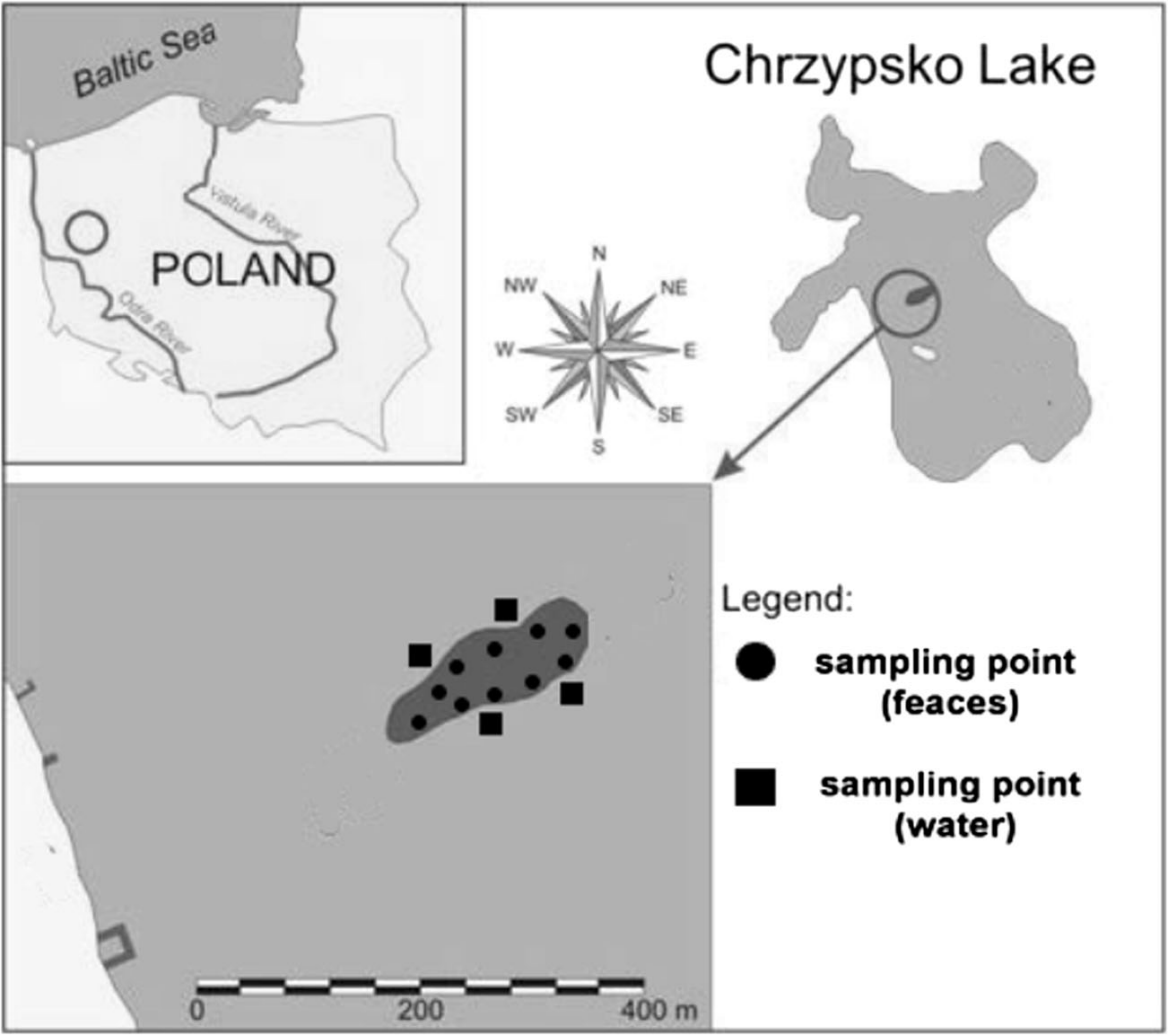
The studied island on Lake Chrzypsko (Poland) inhabited by cormorants and sampling points

The studied colony inhabits the Lake Chrzypsko (Poland, Europe) which is in the state of moderate eutrophy (Klimaszyk 2012). Owing to its location, the lake is intensely used for recreation. Numerous holiday resorts and bathing places are located on its shores. In the west bay, there is also a rowing training center, the racetrack of which stretches near the cormorant colony. The colony has been existing since the beginning of the twenty-first century (Klimaszyk 2012). It occupies the most northward island of the lake (Fig. 1) at the latitude and longitude of 52^o^ 36′ 57″ N and 16^o^13′ 23″ E, respectively. An island has an area of 0.9 ha and slight elevation, up to 40 cm above the lake level. The counting of birds was performed by two independent observers during dawn-dusk prior to collection of fecal samples. During the investigated period (June 2013), 170 breeding pairs (approx. 600 individuals including adults and rearing chicks) were recorded. The foraging area of cormorants during this period is in the radius of 50 km from the colony, but outside the breeding season, adult birds and younglings may spread across the Europe (Bregnballe et al. 2014).

### Samples collection

Samples of cormorant feces were collected from the colony area in June 2013 using 10 trays (60 × 60 cm) located directly beneath bird nests at various points within the island (Fig. 1). The birds’ behavior was observed from the boat using binoculars and feces were systematically collected from each tray. A special care was taken to avoid collecting the fecal samples from the same nest. Plant detritus (leaves, branches) was removed from trays to minimize contamination of samples. Deposited feces were collected to sterile polypropylene tubes by pooling droppings from five different birds as one sample. All samples were immediately preserved with 20 mL potassium dichromate and transported to the laboratory in a lightproof insulated box containing a cooling factor. A total number of 25 pooled samples, and estimated droppings from 125 cormorants, were collected for subsequent parasitological examination. Additionally, samples of lake water (10 L each) were collected from four sampling sites at littoral area near the colony (shore zone) into sterile vessels (Fig. 1). These sites were selected because the shore lake zone was previously shown to be characterized by a high density of fecal bacteria originating from cormorant species (Klimaszyk and Rzymski 2013a; Klimaszyk et al. 2015b).

### Parastiological examination of feces

All fecal samples were examined using coprological methods. From each pooled fecal sample, four smears were made. One direct wet smear was immediately microscopically examined under high dry power (total magnification ×400). The remaining three smears were stained with either: (*i*) modified Weber’s chromotrope 2R–based trichrome stain for *Microsporidia* spores (Weber et al. 1992), (*ii*) Ziehl-Neelsen stain for *Cryptosporidium* oocysts, or (*iii*) iron hematoxylin stain for cysts of *Giardia* and *Blastocystis* (Garcia 2001). Stained smears were microscopically screened using an oil-immersion objective (total magnification ×1000).

Additionally, to confirm identification of *Cryptosporidium* oocysts and/or *Giardia* cysts, all positive specimens were tested using a direct immunofluorescence antibody (IFA) test kit, MERIFLUOR *Cryptosporidium*/*Giardia* (Meridian Diagnostic, Cincinnati, Ohio, USA), was used according to the manufacturer’s instructions.

### Parastiological examination of water samples

All water samples were examined using modified U.S. Environmental Protection Agency Method 1623 (U.S. Environmental Protection Agency 1999). The sediment was obtained by filtration using SM 16274 filter chamber (Sartorius, Germany) on cellulose acetate membranes with a nominal pore size of 0.8 μm (Merck Millipore, Ireland). The filters were then dissolved in acetone according to Graczyk et al. 1997. Each sample concentrate was analyzed using the Ziehl-Neelsen (*Cryptosporidium* oocysts), modified Weber’s chromotrope 2R-based trichrome stain (*Microsporidia* spores) and iron hematoxylin (*Giardia* and *Blastocystis* cysts) methods, and immunofluorescent assay (IFA).

## Results

**Fig. 2:**
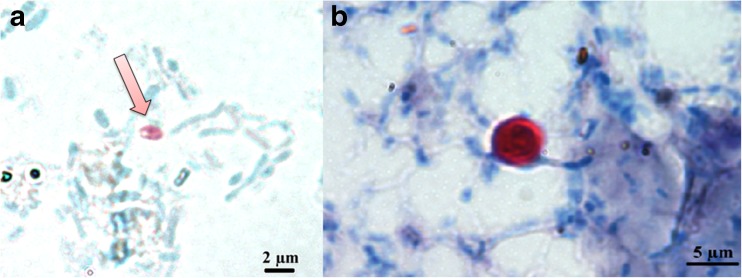
Spores of *Microsporidia* stained with modified Weber’s chromotrope 2R-based trichrome (**a**) and oocysts of *Cryptosporidium* stained with Ziehl-Neelsen (**b**), identified in *Phalacrocorax carbo* feces

*Cryptosporidium* oocysts were identified in 2/25 (8%) of pooled fecal samples of great cormorant (Fig. 2a). All samples detected as positive by Ziehl-Neelsen staining were also positive by the immunofluorescence technique. In both *Cryptosporidium*-positive samples, a small number of oocysts, i.e., five to ten per slide, were detected, at frequency of 3 × 10^3^/g and 5 × 10^3^/g of feces. The mean length (±SD) and width (±SD) of identified oocysts was 5.0 (±0.0) and 5.4 (±0.5) μm, respectively. Spores of *Microsporidia* (Fig. 2b) were identified only in 1/25 (4%) of pooled fecal samples but at high concentration of 4.3 × 10^4^/g of feces. The mean length (±SD) and width (±SD) of these spores was 1.8 (±0.4) and 1.2 (±0.2) μm, respectively. None of the investigated pooled fecal samples contained detectable cysts of *Giardia* and *Blastocystis*.

None of investigated water sample was identified to contain microsporidian spores and dispersive stages of *Cryptosporidium*, *Blastocystis* and *Giardia*.

## Discussion

The birds represent an important factor harboring and dispersing the microorganisms, including pathogens (Graczyk et al. 1998; Okulewicz 2014). The bird microbiota has been demonstrated to be affected by many different factors, such as infections and general health status, diet, and local microbial communities in environment (Palmgren et al. 1997; Lu et al. 2003; Santos et al. 2012). A main route through which the birds can take part in the dispersion of various microorganisms, including protozoa and microsporidia, is fecal excretion. Despite that great cormorants were previously reported to deposit large amounts of feces within the colonized areas (Marion et al. 1994; Klimaszyk and Rzymski 2016), the present study indicates that their role in dispersion of intestinal protozoan parasites may be largely limited and decidedly lower than theoretically expected. Some colonies of this bird may, however, still represent a source of dispersion of other human pathogens (Table 1).

It is important to fully elucidate the biological vectors of dispersive stages of protozoan and microsporidian parasites. These microorganisms are resistant to various environmental conditions, can lead to serious, acute gastrointestinal infections in human, and are usually characterized by the low infectious dose (Szumowski and Troemel 2015; Messner and Berger 2016). The presence of *Cryptosporidium* oocysts and *Giardia* cysts in source waters have already caused numerous documented outbreaks related in both drinking and recreational waters (Karanis et al. 2007). Various birds have already been demonstrated to contribute to contamination of surface waters with dispersive stages of these parasites, including species and genotypes representing a threat to human health (Smith et al. 1993; Graczyk et al. 1998; Majewska et al. 2009). In a Hungarian survey investigating feces of different bird species, one *Giardia* sp. cyst was identified microscopically and the presence of *Cryptosporidium* sp. was confirmed with PCR but the study examined only a single fecal sample collected from cormorant (Plutzer and Tomor 2009). *Cryptosporidium* oocysts (but not *Giardia* cysts) were also detected in feces collected from ten *P. carbo* individuals in the Netherlands inhabiting areas nearby man-made human reservoirs. The prevalence amounted to 20% and mean concentration in positive samples was estimated at 64 oocysts per gram—high enough to significantly contribute to the contamination of water reservoir (Medema 1999). Contrary to these findings, the present study, which employed fecal samples collected from significantly greater number of birds (125 individuals) found that the prevalence of *Cryptosporidum* oocysts was very low and no *Blastocystis* or *Giardia* cysts were present. This indicate that this species, at some inhabited sites, may not represent a significant source of dispersive stages of human protozoan parasites—particularly if one considers that none of investigated protozoans were identified in lake water near the colony.

The infection of the gastrointestinal tract by *Microsporidia* can also lead to severe, persistent diarrhea (Didier 2005). As shown, microsporidian species known to infect humans such as *E. hellem* are present in aquatic bird species including *Anas platyrhynchos*, *Anser anser*, *Balearic pavonina*, *Cygnus atratus*, *C. melanocoryphus*, *C. olor* and *Coscoroba coscoroba* (Słodkowicz-Kowalska et al. 2006). The *E. cuniculi* (but not *E. hellem*, *E. intestinalis* or *E. bieneusi*) was found in the Slovakian pilot study examining 40 samples of great cormorant feces at a relatively high prevalence of 42.5% (Malčeková et al. 2013). The present study showed decidedly lower frequency of spores in investigated population of 125 birds indicating that the role of great cormorants in *Microsporidia* dispersion may be highly site specific. However a relatively high number of spores were identified in one pooled sample (4.3 × 10^4^/g); no dispersive stages of *Microsporidia* were identified in lake water near the colony. It should be highlighted that however the most widely used staining method to detect spores (chromotrope 2R modified trichome) was employed in the present study, it does not allow to distinguish particular species or genotypes of microsporidia. This is important if one considers that only some species (at least 15 from over 1200 identified so far) are known to be pathogenic for humans (Ramanan and Pritt 2014). These, in turn, can be identified by means of immunofluorescence assays using polyclonal or monoclonal antibodies and/or PCR (Ramanan and Pritt 2014). Further investigations are required to fully elucidate the environmental conditions contributing to the presence of microsporidian dispersive stages in cormorant feces, and to estimate risks for human health.

The present study was limited only to one great cormorant colony situated at the lake of low human pressure; therefore, the results should be treated cautiously upon any extrapolation. Sewage discharge can lead to increased contamination of water with dispersive stages of parasites and their presence in biota including waterfowls (Słodkowicz-Kowalska et al. 2015); thus, the prevalence of studied parasites may be different at sites varying in human pressure. It should be, however, highlighted that great cormorants usually nest within areas of negligible human impact (Klimaszyk and Rzymski 2016).

## Conclusions

The present study investigated the presence of dispersive stages of potentially zoonotic protozoans belonging to the genera *Blastocystis*, *Cryptosporidium* and *Giardia*, and *Microsporidia* spores in feces of great cormorant. It was hypothesized that due to specific behavior and metabolism, these birds may represent an important vector for these parasites. Contrary to this, the prevalence of *Cryptosporidium* oocysts and microsporidian spores was very low, and no cysts of *Giardia* and *Blastocystis* were identified. The study indicates that this species may not play, at least at certain locations, a profound role in the dissemination of investigated parasites. Further research employing immunological and molecular methods is necessary to elucidate exact species of *Microsporidia*, and evaluate whether cormorants may disseminate those associated with human infection.
